# Identification of *Neopestalotiopsis* spp. from Strawberry Leaf, Fruit, and Crown Tissues in North Carolina

**DOI:** 10.3390/pathogens15010010

**Published:** 2025-12-21

**Authors:** Swarnalatha Moparthi, Michael J. Bradshaw, William Cline, Michael J. Munster, Mark Hoffmann, Diana Ramirez Segovia, Uma Crouch, Chunying Li, Christie Almeyda, Jhoselin Salazar, Matthew A. Bertone

**Affiliations:** 1 Department of Entomology and Plant Pathology, North Carolina State University, Raleigh, NC 27695, USA; mjbradsh@ncsu.edu (M.J.B.); bill_cline@ncsu.edu (W.C.); mjmunste@ncsu.edu (M.J.M.); diana.ramirezsegovia@gmail.com (D.R.S.); utcrouch@ncsu.edu (U.C.); cli2@ncsu.edu (C.L.); cvalmeyd@ncsu.edu (C.A.); jssalaz2@ncsu.edu (J.S.); maberto2@ncsu.edu (M.A.B.); 2 Department of Horticultural Science, North Carolina State University, Raleigh, NC 27695, USA; mhoffma3@ncsu.edu; 3 Departamento de Protección Vegetal, Universidad de El Salvador, San Salvador 1101, El Salvador; 4 Departamento de Fitopatologia, Universidad Nacional Agraria La Molina, Lima 15024, Peru

**Keywords:** *Neopestalotiopsis* spp., strawberry, North Carolina, greenhouse studies

## Abstract

North Carolina is a leading fresh-market strawberry producer in the southeastern United States, with increasing cultivation driven by consumer demand. In recent years, Neopestalotiopsis-associated diseases have emerged as a major threat to strawberry production, yet limited information is available on their distribution, species diversity, and pathogenic variation in the region. This study investigated the occurrence and characterization of *Neopestalotiopsis* species associated with strawberry crown rot samples submitted to the North Carolina State University Plant Disease and Insect Clinic during 2023–2024. Crowns of diseased plants collected from 27 counties, representing 17 cultivars, were cultured. *Neopestalotiopsis* was the predominant genus (*n* = 114), represented by *N. hispanica* (*n* = 67), species from the *N. rosae* complex (*n* = 44), *N. clavispora* (*n* = 1), *N. scalabiensis* (*n* = 1), and *N. longiappendiculata* (*n* = 1). Greenhouse pathogenicity assays confirmed that the tested *Neopestalotiopsis* isolates were able to cause disease on the strawberry cultivar ‘Fresca’. These findings provide the first comprehensive overview of *Neopestalotiopsis* species associated with strawberry crown rot in North Carolina and highlight their genetic and pathogenic diversity, contributing to improved understanding and management of this emerging disease.

## 1. Introduction

Strawberries (*Fragaria* × *ananassa* Duchesne ex Weston) are herbaceous perennial plants in the family Rosaceae, widely cultivated for their sweet, flavorful, and nutrient-rich fruits [1]. They provide an excellent source of vitamin C, dietary fiber, and antioxidants such as anthocyanins and ellagic acid [2]. Although perennial by nature, strawberries are commonly grown as annual crops in the United States, particularly in regions using plasticulture systems [3]. Successful cultivation requires well-drained soils, moderate temperatures, and careful irrigation management to maintain fruit quality and yield [4]. Because of their high perishability, strawberries demand intensive management and rapid postharvest handling to preserve market quality [5]. In addition, strawberries are sensitive to abiotic stresses such as frost, drought, and heat, which can significantly affect flowering, fruit set and overall productivity [6]. Historically, the cultivated strawberry originated in Europe in the 18th century as an accidental hybrid between the North and South American wild strawberry [7,8]. Since then, it has been widely adapted for global cultivation and has become an economically important crop [9].

In 2023, the United States produced approximately 1.25 million tons of strawberries, making it the second-largest producer globally after China [10]. Within the U.S., California dominates production, contributing more than 85–90% of the total supply [11], while other important states include Florida, Oregon, North Carolina, and Washington [12]. In North Carolina, the majority of the crop is sold directly to consumers through “U-pick” farms and local markets, enhancing its value as a specialty crop [12]. Production relies largely on short-day cultivars such as ‘Chandler’ and ‘Camarosa,’ which are well adapted to the southeastern U.S. [13]. The harvest season typically extends from April through early June. The economic significance of strawberry production in North Carolina is increasing, with expanding local and regional markets driving the adoption of improved cultivation practices and pest management strategies [12].

Strawberry production is impacted by several major pathogens that can significantly reduce yield and fruit quality. These include *Colletotrichum* species [14], *Botrytis cinerea* [15], *Phytophthora* species [16], *Macrophomina phaseolina* [17], *Fusarium oxysporum* f. sp. *fragariae* [18], *Verticillium dahliae* [19] and *Gnomonia* species [20]. Soilborne pathogens, such as *Fusarium oxysporum* f. sp. *fragariae*, *Macrophomina phaseolina* or *Verticillium dahliae* can persist in the soil for multiple seasons, making management particularly challenging [21]. Environmental conditions, especially prolonged humidity and rainfall, can facilitate pathogen spread and increase disease pressure [22]. Moreover, interactions between pathogens and abiotic stressors can further influence disease severity, emphasizing the need for integrated management strategies [23].

In recent years, aggressive *Neopestalotiopsis* species have emerged as a major threat to strawberry production in the southeastern U.S. These pathogens were first confirmed in Florida during the 2018–2019 season and linked to planting material from a North Carolina strawberry nursery [24]. However, in North Carolina fruiting fields, *Neopestalotiopsis* species were first reported in 2022, causing leaf spots, fruit lesions, crown rot, and plant decline resulting in significant yield losses. The most likely route of introduction is through infected planting stock obtained from the nurseries. These plants often appear healthy at the time of planting, allowing the pathogen to spread unnoticed. In North Carolina’s annual plasticulture system, rooted tips (plug plants) are typically planted in late September or early October, creating a window for early pathogen establishment [25]. This has important implications for other strawberry-producing regions, emphasizing the need for vigilant monitoring of nursery stock and early symptom detection.

In addition, the host range of *Neopestalotiopsis* spp. extends beyond strawberry; with recent reports identifying apple [26], mango [27], blueberry [28], avocado [29], grapevine [30], and other horticultural crops as new hosts. This expanding host range highlights the potential threat of *Neopestalotiopsis* spp. to diverse crops and suggests a need for broader surveillance and management practices. The ability of *Neopestalotiopsis* isolates to infect multiple hosts, such as both blueberry and strawberry, increases the potential risk of pathogen establishment in new areas and emphasizes the importance of disease awareness beyond strawberry production [28].

The rapid emergence and aggressive nature of *Neopestalotiopsis* spp. have raised serious concerns among growers, extension personnel, and researchers. Using symptomatic strawberry plants submitted to the North Carolina State University’s Plant Disease and Insect Clinic, the current study was conducted to (i) isolate *Neopestalotiopsis* species from leaves, crowns, and fruits, (ii) speciate the recovered isolates using morphological and molecular methods, (iii) determine the aggressiveness of the recovered isolates using the RFLP method and High-resolution melting (HRM) analysis, and (iv) determine the pathogenicity of selected isolates on strawberry in the greenhouse. Understanding the diversity, distribution, and aggressiveness of these pathogens is essential for developing effective management strategies and informing growers about potential risks to crop productivity.

## 2. Materials and Methods

### 2.1. Pathogen Isolation and Morphological Characterization

**Figure 1:**
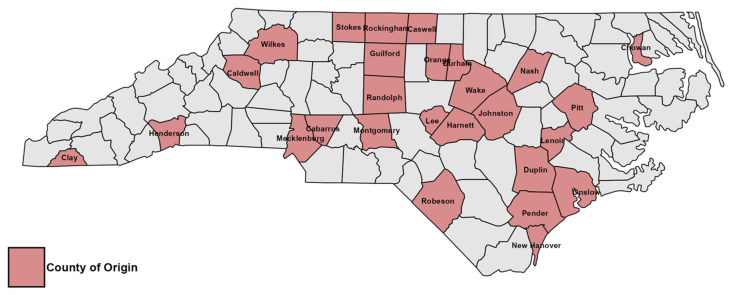
Map of North Carolina showing counties from which *Neopestalotiopsis*-infected strawberry samples were received during the period of this study.

Strawberry plants exhibiting stunting, leaf reddening, and wilting were submitted by growers from 27 counties (Figure 1) across North Carolina in 2023 and 2024 and represented 17 different varieties. No field survey was conducted; all samples were received directly from growers, extension agents or consultants. In the laboratory, roots and crowns were washed with tap water, surface sterilized with 0.6% sodium hypochlorite solution for thirty minutes, rinsed twice with sterile water, and placed on sterile blotting paper in a laminar flow hood. Crown tissue sections were excised from the margins of healthy and symptomatic tissue using a sterile scalpel, and plated onto acidified one-quarter strength potato dextrose agar. This ¼-strength potato dextrose agar (PDA) contained one-quarter of the standard concentration of potato extract and dextrose, with agar added separately for solidification. The medium was prepared by dissolving 9.75 g of PDA powder (MP Biomedicals, Santa Ana, CA, USA) and 11.25 g of granulated agar (Alpha Biosciences Inc., Baltimore, MD, USA) in 1 L of deionized water. After sterilization and cooling to approximately 50 °C, one ml of 50% lactic acid (Thermo Fisher Scientific, Waltham, MA, USA) was added prior to pouring the medium into Petri plates (Fisher Scientific, Suwanee, GA, USA). Quarter-strength agar was used to encourage spore production of *Neopestalotiopsis* species, as regular PDA is high in nutrients. In addition, leaf and fruit samples were incubated in a moist chamber for 2 to 3 days to encourage sporulation. Individual spores were aseptically picked using a needle and placed onto ¼-acidPDA (aPDA). Cultures were then incubated at room temperature (22 °C with minor daily fluctuations) for two to three days. Microscopic examinations involved either placing a culture plate inverted under the microscope or placing conidia on a slide with a drop of distilled water. Plates were incubated at 22 °C for three–five days. Pure cultures were obtained by transferring hyphal tips from colony margins twice onto fresh ¼-aPDA plates.

### 2.2. Genomic DNA Isolation and Amplification

A total of 160 fungal isolates were obtained from strawberry tissues. For each isolate, mycelium (~1–3 mg fresh weight) was collected with a sterile toothpick and transferred into a 2 mL screw-cap extraction tube containing 10 to 20 tiny glass beads (approximately 0.2–0.3 mm in diameter, Thermo Fisher Scientific, Waltham, MA, USA). The samples were vortexed for 30 s to disrupt the mycelium. Genomic DNA was extracted using the PrepMan Ultra kit (Applied Biosystems, Foster City, CA, USA) following the manufacturer’s instructions. DNA concentration and purity were determined using a NanoDrop 2000c spectrophotometer (Thermo Fisher Scientific, Waltham, MA, USA) at 260 nm. The extracted DNA samples were stored at −20 °C until further use. Polymerase chain reactions (PCR) were performed in a thermal cycler (Bio-Rad Laboratories, Hercules, CA, USA) to amplify partial sequences of the translation elongation factor 1-alpha (*TEF*1-α) gene using EF1-728F and EF2 primers [31], β-tubulin gene [32] using Bt2a and Bt2b primers, and internal transcribed spacer (ITS) region using ITS 1 and 4 primers by White et al. [33]. Each 25 µL reaction mixture contained 12.5 µL DreamTaq Green PCR Master Mix (Thermo Fisher Scientific, Waltham, MA, USA), 0.5 µL each of 10 µM forward and reverse primer (IDT primers, Coralville, IA, USA), 9.5 µL nuclease-free water, and 2 µL of template DNA (25 ng/µL). PCR amplification of the ITS region was carried out with an initial denaturation at 94 °C for 4 min, followed by 34 cycles of 94 °C for 45 s, 52 °C for 45 s, and 72 °C for 1 min, with a final extension at 72 °C for 5 min. Amplification of the β-tubulin and *TEF*1-α genes was performed under the same cycling conditions, except that the annealing temperatures were set to 50 °C and 56 °C, respectively. PCR products were visualized on a 1% agarose gel in 1× Tris-acetate-EDTA buffer (AMRESCO, Solon, OH, USA), stained with GelRed™ (Biotium, Fremont, CA, USA), and subsequently sent to Genewiz Inc. (South Plainfield, NJ, USA) for purification and sequencing. Sequences obtained in this study were compared with those in the NCBI GenBank database using BLASTn searches. The sequences were submitted to the GenBank to obtain the accession numbers.

### 2.3. Phylogenetic Analysis

Single-locus phylogenetic trees were constructed from the ITS, β-tubulin and *TEF*1-α regions (Supplementary Figures S1–S3) as well as a concatenated tree (Supplementary Figure S4) of all the sequences in the current study. A concatenated reference tree containing only the six unique *Neopestalotiopsis* genotypes was generated. Taxa were chosen based on the study by Van der Vyver et al. [34]. Sequences were aligned and edited using MUSCLE in MEGA11: Molecular Evolutionary Genetics Analysis Version 11 [35]. A GTR + G + I evolutionary model was used for phylogenetic analyses as it is the most inclusive model of evolution and includes all other evolutionary models [36]. The phylogeny was inferred using Bayesian analysis of the combined loci using a Yule tree prior [37] and a strict molecular clock, in the program BEAST version 1.10.4 [38]. A single MCMC chain of 10^7^ steps was run, with a burn-in of 25%. Posterior probabilities were calculated from the remaining 7500 sampled trees. A maximum clade credibility tree was produced using TreeAnnotator version 1.10.4 (part of the BEAST package). Stationarity was confirmed by running the analysis multiple times, which revealed convergence between runs. The resulting tree was visualized using FigTree ver. 1.3.1 [39]. A maximum likelihood analysis was accomplished using raxmlGUI ver.1.3 [40] under the default settings with a GTR + G + I evolutionary model. Bootstrap analyses were conducted using 1000 replications [41].

### 2.4. Restriction Enzyme Digestion Assay

The DNA region amplified using the primers Bt2a and Bt2b was digested with the restriction enzyme *BsaW*I (New England Biolabs, Beverly, MA, USA), following the protocol described by Kaur et al. [42]. To facilitate comparison with the North Carolina isolates, an aggressive *N. hispanica* isolate (19-02) obtained from the University of Florida was included as a positive control.

### 2.5. High-Resolution Melting (HRM) Analysis

HRM analysis was performed on a few selected isolates (SNrJl01, SNccd02, SNcco03, SNsuj 26, SNsuj46, SNcnd53, SNccrr57, SNsnn60, SNccrl62, SNcrjp63, SNnn78, SNccrw80, SNcm93, SNccm94, SNccm95, SNccr112, SNcrjr114, SNYh127, and SNsp129) following the method described by Rebello et al. [43] for rapid detection and differentiation of *Neopestalotiopsis* species associated with strawberry. The assay targeted polymorphisms within the partial β-tubulin (β-tub)gene region using the primer sets Neo_Tub2_A1F/Neo_Tub2_A1R and Neo_Tub2_B1F/Neo_Tub2_B1R. Quantitative PCR was performed on a QuantStudio 6 Flex Real-Time PCR System (Thermo Fisher Scientific, Applied Biosystems, Waltham, MA, USA), under conditions optimized by Rebello et al. [41]. Representative isolates of *Neopestalotiopsis rosae* (13-481) and *N. hispanica* (19-02) obtained from the University of Florida were included as references. Distinct melting profiles were analyzed to determine species-level differentiation among isolates. All HRM assays were performed in triplicate.

### 2.6. Whole-Plant Pathogenicity Studies in the Greenhouse

A subset of 10 isolates, representing all *Neopestalotiopsis* species identified in this study, was selected for pathogenicity testing. The tests were conducted in a greenhouse using strawberry cv. ‘Fresca’, a seed-propagated cultivar chosen due to the limited availability of commercial cultivars at the time of the experiment. The seedlings were obtained from Ball ColorLink (Ball Horticultural Company, Chicago, IL, USA). Plastic pots (0.5 gallon) were pretreated with 10% sodium hypochlorite (NaOCl) solution and thoroughly rinsed. The pots were then filled with a soil mixture to 5 cm below the rim, and a single seedling was planted in each pot. The growth medium consisted of a 1:1 mixture of peat and Sunshine Mix 1 (Sun Gro Horticulture Inc., Bellevue, WA, USA). Selected *Neopestalotiopsis* isolates (Supplementary Table S1) were cultured on ¼-strength aPDA to prepare inoculum plugs for the greenhouse pathogenicity tests. Agar plugs, 4.5 mm in diameter, were cut from the colony margins of 7-day-old cultures using a cork borer. Two plugs were placed on either side of each seedling in a “sandwich” configuration and covered with Sunshine Mix 1. Agar plugs without fungus served as the negative control. Each treatment consisted of six replicates, with one plant per replicate, arranged in a randomized complete block design. The entire experiment was repeated once. Plants were watered daily and grown on greenhouse benches without supplemental nutrition, maintained at 22 °C during the day and 18 °C at night, under 16:8 h light-dark cycles and ~75% relative humidity. Strawberry plants were harvested 30 days post inoculation, and roots were washed under low-pressure running water. The roots and crowns were then examined for the presence or absence of rot.

### 2.7. Detached-Leaf Pathogenicity Tests

*Neopesalotiopsis hispanica* isolate SNsuj32 and *N. scalabiensis* isolate SNYh127 were grown on ¼-aPDA for 15 days at 20 °C with a 12 h light/12 h dark cycle. The isolates were tested for their ability to cause lesions on leaves of the commercial strawberry cultivars ‘Chandler,’ and ‘Merced.’ Each cultivar was tested in a separate experiment. Leaf inoculation was conducted as mentioned by Baggio et al. [24] except that instead of using an atomizer, inoculum was delivered by pipetting 10 µL of conidial suspension (1 × 10^5^ mL^−1^) onto the adaxial surface of the leaflet. Control leaflets received 10 µL of sterile distilled water. Six leaflets were used in the inoculation per isolate. The leaflets were placed in a moist chamber and incubated at 21 °C with a 12 h photoperiod. The leaflets were observed daily for symptom development.

## 3. Results

### 3.1. Prevalence of Neopestalotiopsis Species and Morphological Characteristics

Of the 160 isolates recovered during the 2023–2024 season, 114 were *Neopestalotiopsis* spp. (71.3% of the total). Other taxa were also detected, including *Fusarium* spp. (8.1%), *Colletotrichum* spp. (5.6%), and several oomycetes (8.8%), with *Phytophthora* spp. being the most frequently recovered. Among the *Neopestalotiopsis* isolates, the highest number were recovered from crowns (82 isolates), followed by leaves (33 isolates) and fruits (18 isolates). Phylogenetic analysis resolved the *Neopestalotiopsis* isolates into five species. *Neopestalotiopsis hispanica* was the predominant species (*n* = 67; 59%), followed by *N. rosae* (*n* = 44; 39%), with single isolates of *N. scalabiensis*, *N. longiappendiculata*, and *N. clavispora* (Supplementary Table S1). The accession numbers for ITS and partial sequences of the β-tubulin and *TEF*1-α genes were obtained from GenBank and are provided in Table 1.

**Figure 2:**
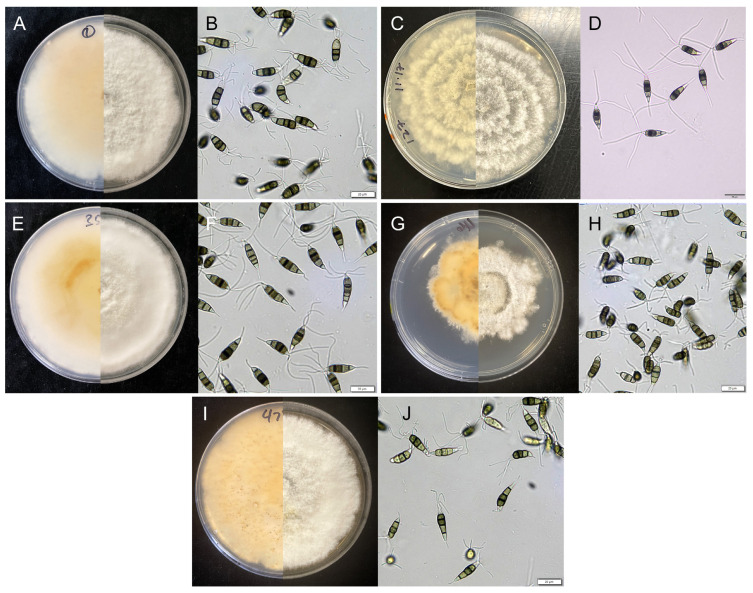
*Neopestalotiopsis rosae* isolate SNrjl01 morphological characteristics on ¼-aPDA (**A**) conidial morphology (**B**). *N. scalabiensis* isolate SNYh127 (**C**) conidial morphology (**D**). *N. hispanica* isolate SNsfj35 (**E**) conidial morphology (**F**). *N. clavispora* isolate SNsuj46 (**G**) conidial morphology (**H**). *N. longiappendiculata* isolate SNccrl62 (**I**) conidial morphology (**J**). Scale bars = 20 µm.

For *Neopestalotiopsis* isolates, conidia were produced within six days after transferring to ¼-aPDA. In all five species, conidiomata were pycnidial, globose, to subglobose, solitary or aggregated, producing dark brown to black conidia in a globose mass. The conidiomata were visible to the naked eye as circular spots on the agar surface (Figure 2). The conidia in all five species were ellipsoid, straight to slightly curved, typically four-septate, though some of the *N. hispanica* isolates have five septa in some of the conidia. The color of the median cells were darker in all five species. All conidia had a single basal appendage and three apical appendages. The apical appendages of *N. hispanica*, *N. longiappendiculata* and *N. scalabiensis* were longer compared to the other two species. The conidial measurements of the five species (*n* = 20) were as follows: *N. clavispora* conidia measured 20 (24) 30 × 5.2 (6.1) 7.7 µm; *N. hispanica* conidia measured 22.7 (27) 31.3 × 5.5 (6.6) 7.9 µm; *N. longiappendiculata* conidia measured 29 (24) 20 × 5.3 (6.5) 8.2 µm; *N. rosae* conidia measured 20 (24) 32 × 5.6 (7.1) 9.1 µm; *N. scalabiensis* conidia measured 22 (25) 29 × 6.4 (7.1) 8.0 µm.

### 3.2. Restriction Fragment Length Polymorphism

RFLP analysis using *BsaW*I generated a distinct double-band pattern (~290 bp and ~130 bp) only for *N. hispanica* isolates and the single isolate of *N. longiappendicualta*. In contrast, *N. rosae*, *N. clavispora*, and *N. scalabiensis* isolates produced an undigested single-band of 420 bp.

### 3.3. High-Resolution Melting Analysis

HRM analysis produced distinct melting-curves for *N. rosae* (primer A: 78.5 °C; primer B: 82.7 °C) and *N. hispanica* (primer A: 79.7 °C; primer B: 81.5 °C). All isolates of these two species from North Carolina showed melting curves consistent with the corresponding positive controls from the University of Florida. For *N. clavispora*, the melting curve generated with primer pair A matched the profile of *N. hispanica*, while the curve produced with primer pair B aligned with that of *N. rosae*. In addition, the melting curve of *N. scalabiensis* was indistinguishable from that of *N. rosae*, and *N. longiappendiculata* showed melting curve similar to *N. hispanica*. The results were consistent across replicates.

### 3.4. Phylogenetic Analysis

**Figure 3:**
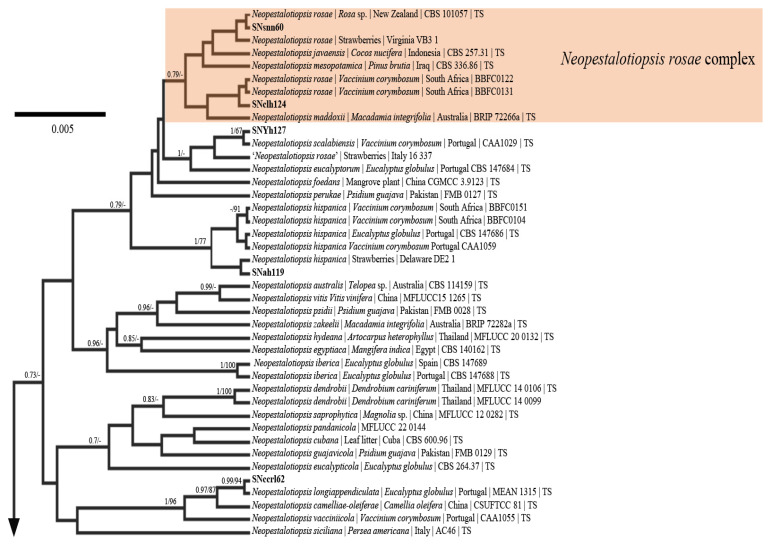

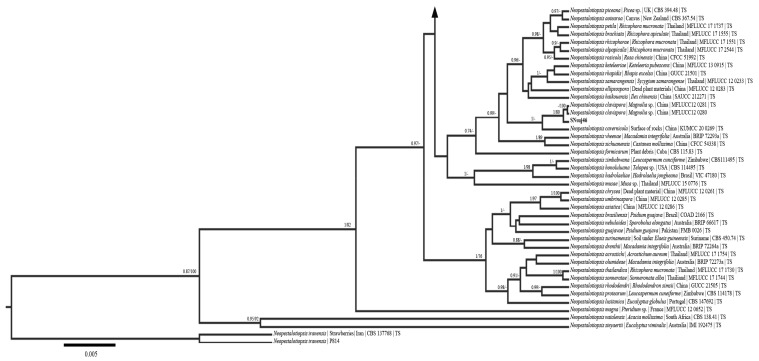
Bayesian maximum clade credibility tree of the concatenated ITS + *TEF*1-α + *TUB*2 regions of select *Neopestalotiopsis* species. Only the six unique *Neopestalotiopsis* genotypes was generated and is shown. There is no support to differentiate *N. rosae*, *N. javaensis*, *N. mesopotamica*, and *N. maddoxii*. Posterior probabilities ≥ 0.70 are displayed followed by bootstrap values greater than 70% for the maximum likelihood (ML) analyses. Taxa in bold were sequenced for the current study. Type specimens are signified with a ‘TS’.

The phylogenetic analyses presented (Figure 3, Supplementary Figures S1–S4) revealed that 6 different genotypes belonging to 5 different species were found to be infecting strawberries in North Carolina. Sequence data included ITS, partial regions of *TEF*1-α + *TUB*2, which were concatenated to resolve relationship among the isolates. There was no support to differentiate *N. rosae*, *N. javaensis*, *N. mesopotamica*, and *N. maddoxii* and, these species were therefore grouped together as the ‘*N. rosae* complex.’ The majority of isolates sequenced were identified as *N. hispanica* followed by isolates within the *N. rosae* complex. Single isolates were identified as belonging to *N. scalabiensis*, *N. longiappendiculata*, and *N. clavispora*. Across all phylogenetic trees, isolates clustered according to species, with *N. hispanica* forming a distinct branch separate from the *N. rosae* complex and the other single-isolate species. The branching patterns were consistent across all three regions analyzed, and sequences from the concatenated dataset reinforced the species-level clustering observed in the individual gene trees.

### 3.5. Pathogenicity Tests

**Figure 4:**
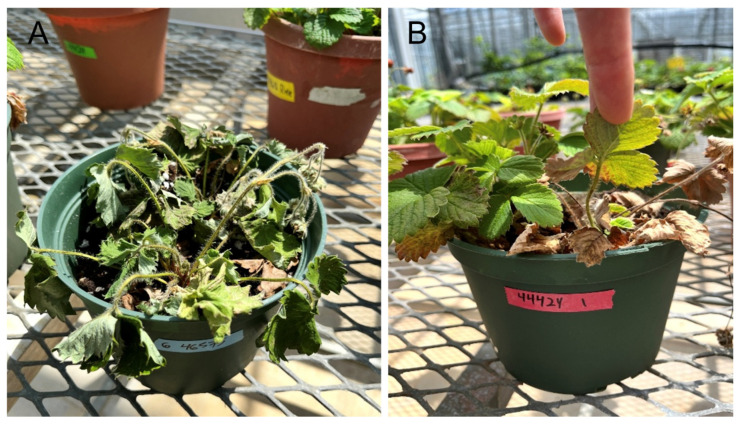
Symptoms observed on strawberry cultivar ‘Fresca’ during greenhouse inoculation experiments. (**A**) Strawberry cultivar ‘Fresca’ displaying wilting symptoms 25 days after inoculation with *N. hispanica* isolate SNsfj35 (**B**) Strawberry cultivar ‘Fresca’ displaying symptoms 25 days after inoculation with *N. rosae* isolate SNrjl01.

Root and crown rot symptoms were evident on greenhouse-inoculated strawberry plants 30 days after inoculation. Infected plants exhibited varying degrees of symptom severity, ranging from mild root browning to extensive crown tissue decay and wilting (Figure 4). In several cases, the crowns were discolored and softened, and the root systems were noticeably reduced compared to non-inoculated controls. A few plants appeared healthy and showed no visible signs of rot, but overall, the incidence of inoculated asymptomatic plants was low. Infection was observed in multiple plants within each replicate, indicating successful establishment of disease under greenhouse conditions. *Neopestalotiopsis* spp. were successfully recovered from these tissues, and the colony morphology and microscopic characteristics were consistent with those of the inoculated *Neopestalotiopsis* isolates, confirming that the observed symptoms were caused by the species being tested.

**Figure 5:**
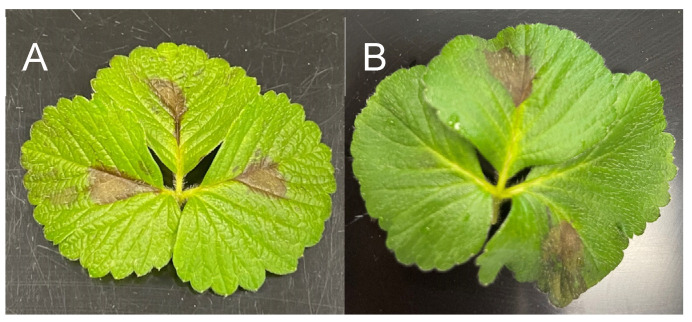
Symptoms in detached leaf assays six days post-inoculation with *N. hispanica* SNsfj35 on strawberry cultivars ‘Chandler’ (**A**) and ‘Merced’ (**B**).

In the detached leaf assays, leaves inoculated with spore suspensions of *N. hispanica* and *N. scalabiensis* developed characteristic lesions within six days. The lesions initially appeared as small, light-brown to purple spots on the adaxial surface within two days after inoculation and gradually expanded, coalesced, and became necrotic over time (Figure 5). Symptom development was consistent among replicated leaflets and followed a similar progression for both species. Control leaflets, which received sterile water, remained free of symptoms throughout the observation period, confirming that lesion development was attributable to fungal infection. Pycnidia developed within the lesions in concentric rings approximately two weeks after inoculation, indicating active sporulation on infected tissue. Collectively, these results confirmed that the isolates tested were capable of infecting leaf tissue and inducing disease symptoms under controlled conditions without the need for wounding.

## 4. Discussion

The current study demonstrates that five phylogenetically distinct species of *Neopestalotiopsis* are associated with strawberries in North Carolina. Among these, *N. hispanica* was detected at a notably higher frequency than the other species followed by isolates from the *N. rosae* complex. In the current study we introduced the term *N. rosae* complex because there was minimal support to separate the nominate species from closely related species in all of our analyses (Supplementary Figures S1–S3, Figure 3). These results are similar to those of Vand der Vyer et al. [34] whose analyses also exhibited minimal support for these species. Future research will need to apply additional markers and analyze the morphology of the specimens concerned to determine if this is indeed a complex of species, or a single, morphologically similar species with a wide host range.

Similar to our study, a recent work from Florida reported a high diversity of *Neopestalotiopsis* species from strawberry leaves and fruits [24]. These species have since been reported from multiple U.S. states, including South Carolina [42], Georgia [44], and Delaware [45]. In contrast, *N. rosae* has been documented causing disease on strawberries in California [46] and Italy [47] but those isolates could not be confirmed to fall in the *N. rosae* complex in the current paper.

The higher number of isolates recovered from crown tissues reflects the type of samples received by the diagnostic clinic rather than the true distribution of *Neopestalotiopsis* infections in the field. Whole plants are more commonly submitted than individual leaf or fruit samples, which naturally results in more crown-associated isolates being recovered. Nevertheless, the predominance of *N. hispanica* across the submissions indicates that this species is frequently associated with symptomatic strawberry plants in North Carolina and continues to be the most commonly encountered species in diagnostic samples. These findings highlight areas of higher pathogen prevalence, providing a basis for targeted surveillance. Knowledge of which species predominate in specific counties (or from specific nursery source) can inform growers about potential risk factors, such as cultivar susceptibility, planting density, or cultural practices, and guide implementation of integrated disease management strategies.

*Neopestalotiopsis vaccinii* was recently reported from Delaware as a pathogen of strawberry [45]; however, Van der Vyer et al. [34] have synonymized this species with *N. hispanica*. Isolates from the *N. rosae* complex, although less frequent, were also recovered from multiple submissions, suggesting that it may play a less frequent but notable role in disease expression. In contrast, *N. clavispora*, *N. scalabiensis*, and *N. longiappendiculata* were detected only once each. While *N. clavispora* has been reported previously on strawberry, from Spain [48], India [49], Italy [50], and China [51], to date, there are no published reports of *N. longiappendiculata* or *N. scalabiensis* associated with disease in strawberry. This suggests that these species may be rare or under-recognized pathogens in this crop.

The morphological features observed in culture are consistent with species descriptions by Baggio et al. [24], including rapid conidial production on artificial media and the formation of dark, globose pycnidial conidiomata. Interestingly, some of our *N. hispanica* isolates produced conidia with five septa, a feature not reported in the standard descriptions of this species; to date, *N. hispanica* has been consistently described as having four-septate conidia. Our observations may represent a novel morphological variant. To verify this, further work is needed, including sporulation under varied culture conditions, quantifying septation frequency, and extended phylogenetic analysis using additional loci. Morphological traits, such as conidial length and width, apical appendage length, the presence or absence of knobbed appendages, and the position of the apical appendage on the conidial body vary among *Neopestalotiopsis* species [52]. However, conidial dimensions and morphology often overlap and so are not sufficient for reliable species-level identification; molecular phylogenetic analysis is indispensable [52,53].

The RFLP analysis using *BsaW*I proved effective for identifying *N. hispanica* and *N. longiappendiculata*, as both species produced the same distinct double-band pattern consistent with previous reports [42]. However, this method did not allow discrimination between these two species, nor did the method differentiate aggressive from non-aggressive isolates of *N. rosae*, *N. clavispora*, and *N. scalabiensis*, highlighting a key limitation of this approach. The HRM assay was able to reliably differentiate *N. rosae*, *N. clavispora*, and *N. hispanica* based on their melting-curve profiles. When *N. scalabiensis* and *N. longiappendiculata* were included, the assay could not distinguish these species from the others, reflecting sequence similarities in the region targeted by the primers. Previous work by Rebello et al. [43] likely focused on separating *N. rosae* and *N. hispanica* and would not have included the additional *Neopestalotiopsis* species, as our study is the first to report three additional species associated with strawberry. Our results indicate that both RFLP and HRM methods, which rely on the beta-tubulin gene, are unable to distinguish some closely related or newly described species, because sequences such as those of *N. longiappendiculata* and *N. hispanica* share identical base pairs in this region. Future experiments should incorporate these newly identified species and other *Neopestalotiopsis* species to fully assess the utility of HRM for differentiating all strawberry-associated *Neopestalotiopsis* species.

The successful infection of plants from the cultivar ‘Fresca’ in the greenhouse and leaves of the cultivars ‘Chandler’ and ‘Merced’ under controlled conditions, demonstrates that the *Neopestalotiopsis* isolates recovered from North Carolina strawberries are highly aggressive across plant tissues. We recognize that susceptibility of a seed-propagated cultivar like ‘Fresca’ may differ from that of vegetatively propagated commercial cultivars, but the latter were unavailable when the whole-plant inoculations were performed. It is notable that our leaf- and fruit- recovered *Neopestalotiopsis* isolates caused crown symptoms. This observation is consistent with recent reports showing that *N. rosae* can infect various parts of the strawberry plant, highlighting the pathogen’s potential to cause widespread damage within the plant. These findings underscore the need for comprehensive monitoring of all plant parts in current production and suggest that future research should focus on understanding tissue-specific susceptibility and developing targeted management strategies. For instance, Fernández-Ozuna et al. [54] mentioned *N. rosae* causing both leaf spot and crown rot on strawberry in Paraguay. Integrating these pathogenicity data with species prevalence information can help growers better understand the overall disease risk, prioritize regular monitoring, and implement management practices aimed at reducing pathogen spread, ultimately supporting more effective and sustainable crop protection.

For better disease management, future cross-inoculation trials should include all *Neopestalotiopsis* species to assess their aggressiveness across different strawberry parts and evaluate their potential for systemic infection. Importantly, the isolates tested originated from multiple commercial cultivars, suggesting that these *Neopestalotiopsis* species can infect a broad range of genetic backgrounds and are not limited to a specific cultivar. Ongoing field and greenhouse studies aim to quantify aggressiveness across commercial cultivars, which will be critical for identifying cultivar-specific resistance and for developing effective disease management strategies. This information will also allow breeders to screen cultivars against multiple *Neopestalotiopsis* species and enable fungicide efficacy testing against all aggressive species.

## 5. Conclusions

This study provides the first comprehensive investigation of Neopestalotiopsis disease of strawberry in North Carolina. Over a two-year sampling period, we recovered 114 *Neopestalotiopsis* isolates from symptomatic crown, leaf, and fruit tissues, confirming that this pathogen is the predominant cause of strawberry disease across the state’s major production regions. This study reports *N. clavispora*, and *N. scalabiensis* and *N. longiappendiculata* associated with strawberry in the USA for the first time. The findings underscore the aggressiveness of these pathogens across multiple cultivars, emphasizing the need for effective management strategies. Globally, *Neopestalotiopsis* species have been reported as emerging threats to strawberry production in multiple countries, including Italy, Spain, China and Paraguay, highlighting the international relevance of understanding their diversity, host range, and pathogenicity. Future research should focus on evaluating the sensitivity of different *Neopestalotiopsis* species to commonly used fungicides, determining cultivar-specific susceptibility, and investigating how environmental conditions and cultural practices influence disease development and epidemic dynamics. Such work will be critical for developing integrated disease management practices and mitigating losses in commercial strawberry production.

## Figures and Tables

**Table 1:** List of *Neopestalotiopsis* species, isolate identifiers, and GenBank accessions for ITS, *TEF*1-α, and β-tubulin loci.

		GenBank Accession Number		
*Neopestalotiopsis* spp.	Isolate	ITS	*TEF*1-α	β-tubulin
*N. rosae*	SNcah118	PX563605	PX711530	PX711490
*N. rosae*	SNcc08	PX563606	PX711531	PX711491
*N. rosae*	SNcc09	PX563607	PX711532	PX711492
*N. rosae*	SNcc10	PX563608	PX711533	PX711493
*N. rosae*	SNcc11	PX563609	PX711534	PX711494
*N. rosae*	SNcc117	PX563610	PX711535	PX711495
*N. rosae*	SNccd02	PX563611	PX711536	PX711496
*N. rosae*	SNccd16	PX563612	PX711537	PX711497
*N. rosae*	SNccg04	PX563613	PX711538	PX711498
*N. rosae*	SNccm94	PX563614	PX711539	PX711499
*N. rosae*	SNccm95	PX563615	PX711540	PX711500
*N. rosae*	SNcco03	PX563616	PX711541	PX711501
*N. rosae*	SNcco72	PX563617	PX711542	PX711502
*N. rosae*	SNccr112	PX563618	PX711543	PX711503
*N. rosae*	SNccrr57	PX563619	PX711544	PX711504
*N. rosae*	SNclh124	PX563620	PX711545	NA ^1^
*N. rosae*	SNcm93	PX563621	PX711546	PX711505
*N. rosae*	SNcnc100	PX563622	PX711547	PX711506
*N. rosae*	SNcnc99	PX563623	PX711548	PX711507
*N. rosae*	SNcnd17	PX563624	PX711549	PX711508
*N. rosae*	SNcnd53	PX563625	PX711550	PX711509
*N. rosae*	SNcnd54	PX563626	PX711551	PX711510
*N. rosae*	SNcnd55	PX563627	PX711552	PX711511
*N. rosae*	SNcnd56	PX563628	PX711553	PX711512
*N. rosae*	SNcnh86	PX563629	PX711554	PX711513
*N. rosae*	SNcnn81	PX563630	PX711555	PX711514
*N. rosae*	SNcnrjl52	PX563631	PX711556	PX711515
*N. rosae*	SNco73	PX563632	PX711557	PX711516
*N. rosae*	SNcph125	PX563633	PX711558	NA ^1^
*N. rosae*	SNcrj108	PX563634	PX711559	PX711517
*N. rosae*	SNcrj109	PX563635	PX711560	PX711518
*N. rosae*	SNcrjp63	PX563636	PX711561	PX711519
*N. rosae*	SNcrjp64	PX563637	PX711562	PX711520
*N. rosae*	SNcrjr114	PX563638	PX711563	PX711521
*N. rosae*	SNcscr58	PX563639	PX711564	PX711522
*N. rosae*	SNcscr59	PX563640	PX711565	PX711523
*N. rosae*	SNcw12	PX563641	PX711566	PX711524
*N. rosae*	SNcw13	PX563642	PX711567	PX711525
*N. rosae*	SNar79	PX563643	PX711568	PX711526
*N. rosae*	SNsnn60	PX563644	PX711569	PX711527
*N. rosae*	SNsrjp65	PX563645	PX711570	PX711528
*N. rosae*	SNrjl01	PX563646	PX711571	PX711529
*N. clavispora*	SNsuj46	PX586188	PX699155	PX699157
*N. hispanica*	SNah119	PX619695	PX699089	PX711486
*N. hispanica*	SNah120	PX619696	PX699090	PX711485
*N. hispanica*	SNas104	PX619697	PX699091	PX711484
*N. hispanica*	SNcno51	PX619698	PX699092	PX711487
*N. hispanica*	SNcrh69	PX619699	PX699093	PX711489
*N. hispanica*	SNcrp06	PX619700	PX699094	PX711483
*N. hispanica*	SNcrp07	PX619701	PX699095	PX711482
*N. hispanica*	SNht14	PX619702	PX699154	PX711481
*N. hispanica*	SNmh122	PX619703	PX699096	PX711480
*N. hispanica*	SNmh123	PX619704	PX699097	PX711479
*N. hispanica*	SNnac19	PX619705	PX699098	PX711478
*N. hispanica*	SNnc18	PX619706	PX699099	PX711477
*N. hispanica*	SNnh15	PX619707	PX699100	PX711476
*N. hispanica*	SNnh85	PX619708	PX699101	PX711475
*N. hispanica*	SNnh88	PX619709	PX699102	PX711474
*N. hispanica*	SNnl98	PX619710	PX699103	PX711473
*N. hispanica*	SNnn78	PX619711	PX699104	PX711472
*N. hispanica*	SNnn90	PX619712	PX699105	PX711471
*N. hispanica*	SNnn91	PX619713	PX699106	PX711470
*N. hispanica*	SNnn92	PX619714	PX699107	PX711469
*N. hispanica*	SNnn101	PX619715	PX699108	PX711468
*N. hispanica*	SNnnh83	PX619716	PX699109	PX711467
*N. hispanica*	SNnnh84	PX619717	PX699110	PX711466
*N. hispanica*	SNp132	PX619718	PX699111	PX711465
*N. hispanica*	SNrjh121	PX619719	PX699112	PX711464
*N. hispanica*	SNrn134	PX619720	PX699113	PX711463
*N. hispanica*	SNsajj36	PX619721	PX699114	PX711462
*N. hispanica*	SNsc82	PX619722	PX699115	PX711461
*N. hispanica*	SNscc20	PX619723	PX699116	PX711460
*N. hispanica*	SNscc21	PX619724	PX699117	PX711459
*N. hispanica*	SNscm67	PX619725	PX699118	PX711458
*N. hispanica*	SNscm68	PX619726	PX699119	PX711457
*N. hispanica*	SNscr61	PX619727	PX699120	PX711456
*N. hispanica*	SNscrh70	PX619728	PX699121	PX711455
*N. hispanica*	SNscrh71	PX619729	PX699122	PX711454
*N. hispanica*	SNscrj28	PX619730	PX699123	PX711453
*N. hispanica*	SNscrj34	PX619731	PX699124	PX711452
*N. hispanica*	SNscrj45	PX619732	PX699125	PX711451
*N. hispanica*	SNsfj24	PX619733	PX699126	PX711450
*N. hispanica*	SNsfj35	PX619734	PX699127	PX711449
*N. hispanica*	SNsmjj37	PX619735	PX699128	PX711448
*N. hispanica*	SNsn135	PX619736	PX699129	PX711447
*N. hispanica*	SNsnh87	PX619737	PX699130	PX711446
*N. hispanica*	SNsnj22	PX619738	PX699131	PX711445
*N. hispanica*	SNsnj23	PX619739	PX699132	PX711444
*N. hispanica*	SNso50	PX619740	PX699133	PX711443
*N. hispanica*	SNsp129	PX619741	PX699134	PX711442
*N. hispanica*	SNssj25	PX619742	PX699135	PX711441
*N. hispanica*	SNssn133	PX619743	PX699136	PX711440
*N. hispanica*	SNssuj38	PX619744	PX699137	PX711439
*N. hispanica*	SNssuj39	PX619745	PX699138	PX711438
*N. hispanica*	SNsuj26	PX619746	PX699139	PX711437
*N. hispanica*	SNsuj27	PX619747	PX699140	PX711436
*N. hispanica*	SNsuj29	PX619748	PX699141	PX711435
*N. hispanica*	SNsuj30	PX619749	PX699142	PX711434
*N. hispanica*	SNsuj31	PX619750	PX699143	PX711433
*N. hispanica*	SNsuj32	PX619751	PX699144	PX711432
*N. hispanica*	SNsuj33	PX619752	PX699145	PX711431
*N. hispanica*	SNsuj40	PX619753	PX699146	PX711430
*N. hispanica*	SNsuj41	PX619754	PX699147	PX711429
*N. hispanica*	SNsuj42	PX619755	PX699148	PX711428
*N. hispanica*	SNsuj43	PX619756	PX699149	PX711427
*N. hispanica*	SNsuj44	PX619757	PX699150	PX711426
*N. hispanica*	SNsuj47	PX619758	PX699151	PX711425
*N. hispanica*	SNsuj48	PX619759	PX699152	PX711424
*N. hispanica*	SNucnh05	PX619760	PX699153	PX711486
*N. longiappendiculata*	SNccrl62	PX643237	PX699156	PX699159
*N. scalabiensis*	SNYh127	PX643238	NA ^1^	PX699158

^1^ Accession number not available.

## Data Availability

The sequences (ITS, partial regions of *TEF*1-α, and *TUB*2) used in the study were deposited in the GenBank and the accession numbers are shown in the manuscript.

## Supplementary Materials

The following supporting information can be downloaded at: https://www.mdpi.com/article/10.3390/pathogens15010010/s1, Figure S1: Bayesian maximum clade credibility tree of the ITS region of all the isolates sequenced in the current study as well as select *Neopestalotiopsis* species.; Figure S2: Bayesian maximum clade credibility tree of the *TEF*1-α region of all the isolates sequenced in the current study as well as select *Neopestalotiopsis* species. Figure S3: Bayesian maximum clade credibility tree of the *TUB*2 region of all the isolates sequenced in the current study as well as select *Neopestalotiopsis* species. Figure S4: Bayesian maximum clade credibility tree of the concatenated ITS + *TEF*1-α + *TUB*2 regions of all the isolates sequenced in the current study as well as select *Neopestalotiopsis* species. Table S1: *Neopestalotiopsis* isolates associated with strawberry disease in North Carolina.
