# Supplementary material for: Identification of Neopestalotiopsis spp. from Strawberry Leaf, Fruit, and Crown Tissues in North Carolina
Source: Pathogens. 2025 Dec 21;15(1):10. doi: 10.3390/pathogens15010010 (PMC12844628; doi:10.3390/pathogens15010010)
Supplement: Supplementary file 1 [file pathogens-15-00010-s001.zip › Supplementary table.pdf]

Table S1: *Neopestalotiopsis* isolates associated with strawberry disease in North Carolina.

| Isolate | Isolation source | Strawberry cultivar | County | *Neopestalotiopsis* species |
|---|---|---|---|---|
| **SNrJl01** | crown | Ruby June | Lee | *N. rosae* |
| SNccd02 | crown | Chandler | Durham | *N. rosae* |
| SNcco03 | crown | Chandler | Orange | *N. rosae* |
| SNccg04 | crown | Chandler | Guilford | *N. rosae* |
| SNucnh05 | crown | unreleased | New Hanover | *N. hispanica* |
| **SNcrp06** | crown | Camino Real | Pender | *N. hispanica* |
| SNcrp07 | crown | Camino Real | Pender | *N. hispanica* |
| SNcc08 | crown | NA | Cabarrus | *N. rosae* |
| SNcc09 | crown | NA | Cabarrus | *N. rosae* |
| SNcc10 | crown | NA | Cabarrus | *N. rosae* |
| SNcc11 | crown | NA | Cabarrus | *N. rosae* |
| SNcw12 | crown | NA | Wake | *N. rosae* |
| SNcw13 | crown | NA | Wake | *N. rosae* |
| SNht14 | fruit | NA | Harnett | *N. hispanica* |
| SNnh15 | fruit | NA | Harnett | *N. hispanica* |
| SNccd16 | crown | NA | Duplin | *N. rosae* |
| SNcnd17 | fruit | NA | Duplin | *N. rosae* |
| SNnc18 | fruit | NA | Chowan | *N. hispanica* |
| SNnac19 | leaf | NA | Chowan | *N. hispanica* |
| SNscc20 | crown | Sweet Charlie | Chowan | *N. hispanica* |
| SNscc21 | crown | Chandler | Chowan | *N. hispanica* |
| SNsnj22 | crown | NA | Johnston | *N. hispanica* |
| SNsnj23 | crown | NA | Johnston | *N. hispanica* |
| SNsfj24 | crown | Felicity | Johnston | *N. hispanica* |
| SNssj25 | crown | Sensation | Johnston | *N. hispanica* |
| SNsuj26 | crown | 113 NC 21-033 | Johnston | *N. hispanica* |
| SNsuj27 | crown | 114 NC20- 055 | Johnston | *N. hispanica* |
| SNscrj28 | crown | Camino Real | Johnston | *N. hispanica* |
| SNsuj29 | crown | 205 NC 20-055 | Johnston | *N. hispanica* |
| SNsuj30 | crown | 206 NC 20-099 | Johnston | *N. hispanica* |
| SNsuj31 | crown | 209 NC 19-020 | Johnston | *N. hispanica* |
| SNsuj32 | crown | 210 NC19-023 | Johnston | *N. hispanica* |
| SNsuj33 | crown | 211 NC 22-014 | Johnston | *N. hispanica* |
| SNscrj34 | crown | Camino Real | Johnston | *N. hispanica* |
| **SNsfj35** | crown | Felicity | Johnston | *N. hispanica* |
| SNsajj36 | crown | Ashley Joy | Johnston | *N. hispanica* |
| SNsmjj37 | crown | Miss Joy | Johnston | *N. hispanica* |
| SNssuj38 | crown | 306 NC 20-099 | Johnston | *N. hispanica* |
| SNssuj39 | crown | 307 NC 21-033 | Johnston | *N. hispanica* |

| | | | | |
|---|---|---|---|---|
| SNsuj40 | crown | 308 NC 19-016 | Johnston | *N. hispanica* |
| SNsuj41 | crown | 309 NC 22-014 | Johnston | *N. hispanica* |
| SNsuj42 | crown | 311 NC 20-055 | Johnston | *N. hispanica* |
| SNsuj43 | crown | 314 NC 19-020 | Johnston | *N. hispanica* |
| SNsuj44 | crown | 317 NC 22-005 | Johnston | *N. hispanica* |
| SNscrj45 | crown | Camino Real | Johnston | *N. hispanica* |
| **SNsuj46** | crown | NC 21-031 | Johnston | *N. clavispora* |
| SNsuj47 | crown | NC 22-003 | Johnston | *N. hispanica* |
| SNsuj48 | crown | 512 NC22-001 | Johnston | *N. hispanica* |
| SNsuj49 | crown | 513 NC 21-035 | Johnston | *N. hispanica* |
| SNso50 | crown | NA | Orange | *N. hispanica* |
| SNcno51 | fruit | NA | Orange | *N. hispanica* |
| SNcnrjl52 | crown | Ruby June | Lee | *N. rosae* |
| SNcnd53 | crown | NA | Duplin | *N. rosae* |
| SNcnd54 | crown | NA | Duplin | *N. rosae* |
| SNcnd55 | crown | NA | Duplin | *N. rosae* |
| SNcnd56 | crown | NA | Duplin | *N. rosae* |
| **SNccrr57** | crown | Camarosa | Robeson | *N. rosae* |
| SNcscr58 | crown | Sweet Charlie | Rockingham | *N. rosae* |
| SNcscr59 | crown | Sweet Charlie | Rockignham | *N. rosae* |
| SNsnn60 | crown | NA | NA | *N. rosae* |
| SNscr61 | crown | Chandler | Robeson | *N. hispanica* |
| **SNccrl62** | crown | Camino Real | Lenoir | *N. longiappendiculata* |
| **SNcrjp63** | crown | Ruby June | Pitt | *N. rosae* |
| SNcrjp64 | crown | Ruby June | Pitt | *N. rosae* |
| SNsrjp65 | crown | Ruby June | Pitt | *N. rosae* |
| SNscm67 | crown | Chandler | Montgomery | *N. hispanica* |
| SNscm68 | crown | Chandler | Montgomery | *N. hispanica* |
| SNcrh69 | crown | Camino Real | Harnett | *N. hispanica* |
| SNscrh70 | crown | Camino Real | Harnett | *N. hispanica* |
| SNscrh71 | crown | Camino Real | Harnett | *N. hispanica* |
| SNcco72 | crown | Chandler | Orange | *N. rosae* |
| SNco73 | crown | NA | Onslow | *N. rosae* |
| PNFS76 | NA | NA | NA | *N. hispanica* |
| **SNnn78** | fruit | NA | Randolph | *N. rosae* |
| SNnar79 | crown | Camino Real | Wilkes | *N. rosae* |
| SNccrw80 | crown | NA | NA | *N. rosae* |
| SNcnn81 | leaf | NA | Clay | *N. hispanica* |
| SNsc82 | crown | NA | New Hanover | *N. hispanica* |
| SNnnh83 | leaf | NA | New Hanover | *N. hispanica* |
| SNnnh84 | leaf | NA | New Hanover | *N. hispanica* |
| SNnh85 | leaf | NA | New Hanover | *N. rosae* |
| SNcnh86 | leaf | NA | New Hanover | *N. hispanica* |

| | | | | |
|---|---|---|---|---|
| SNsnh87 | crown | NA | New Hanover | *N. hispanica* |
| SNnh88 | leaf | NA | Nash | *N. hispanica* |
| SNnn90 | leaf | NA | Nash | *N. hispanica* |
| SNnn91 | leaf | NA | Nash | *N. hispanica* |
| SNnn92 | crown | Chandler | Mecklenburg | *N. rosae* |
| SNcm93 | crown | Chandler | Mecklenburg | *N. rosae* |
| SNccm94 | crown | Chandler | Mecklenburg | *N. rosae* |
| SNccm95 | crown | NA | Wake | *N. hispanica* |
| SNnl98 | leaf | NA | Caswell | *N. rosae* |
| SNcnc99 | leaf | NA | Nash | *N. hispanica* |
| SNcnc100 | leaf | Albion | Stokes | *N. hispanica* |
| SNnn101 | crown | Ruby June | Caswell | *N. rosae* |
| SNas104 | crown | Ruby June | Lee | *N. rosae* |
| SNrj105 | leaf | Ruby June | Lee | *N. rosae* |
| **SNcrj108** | leaf | Chandler | Rockingham | *N. rosae* |
| SNcrj109 | crown | Ruby June | Rockingham | *N. rosae* |
| SNcrjr110 | leaf | Chandler | Caldwell | *N. rosae* |
| SNcr111 | crown | Albion | Henderson | *N. rosae* |
| SNccr112 | leaf | Albion | Henderson | *N. hispanica* |
| SNcrjr114 | leaf | Albion | Henderson | *N. hispanica* |
| SNcc117 | leaf | Malwina | Henderson | *N. hispanica* |
| SNcah118 | leaf | Darselect | Henderson | *N. rosae* |
| SNah119 | leaf | Lucille | Henderson | *N. rosae* |
| SNrjh121 | leaf | Yembu | Henderson | *N. rosae* |
| SNmh122 | leaf | Camino Real | Henderson | *N. hispanica* |
| SNmh123 | leaf | Ember | Henderson | *N. hispanica* |
| SNclh124 | leaf | Brilliance | Henderson | *N. hispanica* |
| SNcph125 | leaf | Ember | Henderson | *N. hispanica* |
| SNcmh126 | leaf | Sensation | Henderson | *N. hispanica* |
| **SNYh127** | crown | Yembu | Henderson | *N. scalabiensis* |
| SNsp129 | leaf | Camino Real | Pender | *N. hispanica* |
| SNp132 | leaf | Brillaince | Pender | *N. hispanica* |

*Neopesatlotiopsis* isolates that were used in the greenhouse pathogenicity and detached leaf tests are shown in bold.
