# Supplementary figures and images for: Identification of Neopestalotiopsis spp. from Strawberry Leaf, Fruit, and Crown Tissues in North Carolina
Source: Pathogens. 2025 Dec 21;15(1):10. doi: 10.3390/pathogens15010010 (PMC12844628; doi:10.3390/pathogens15010010)

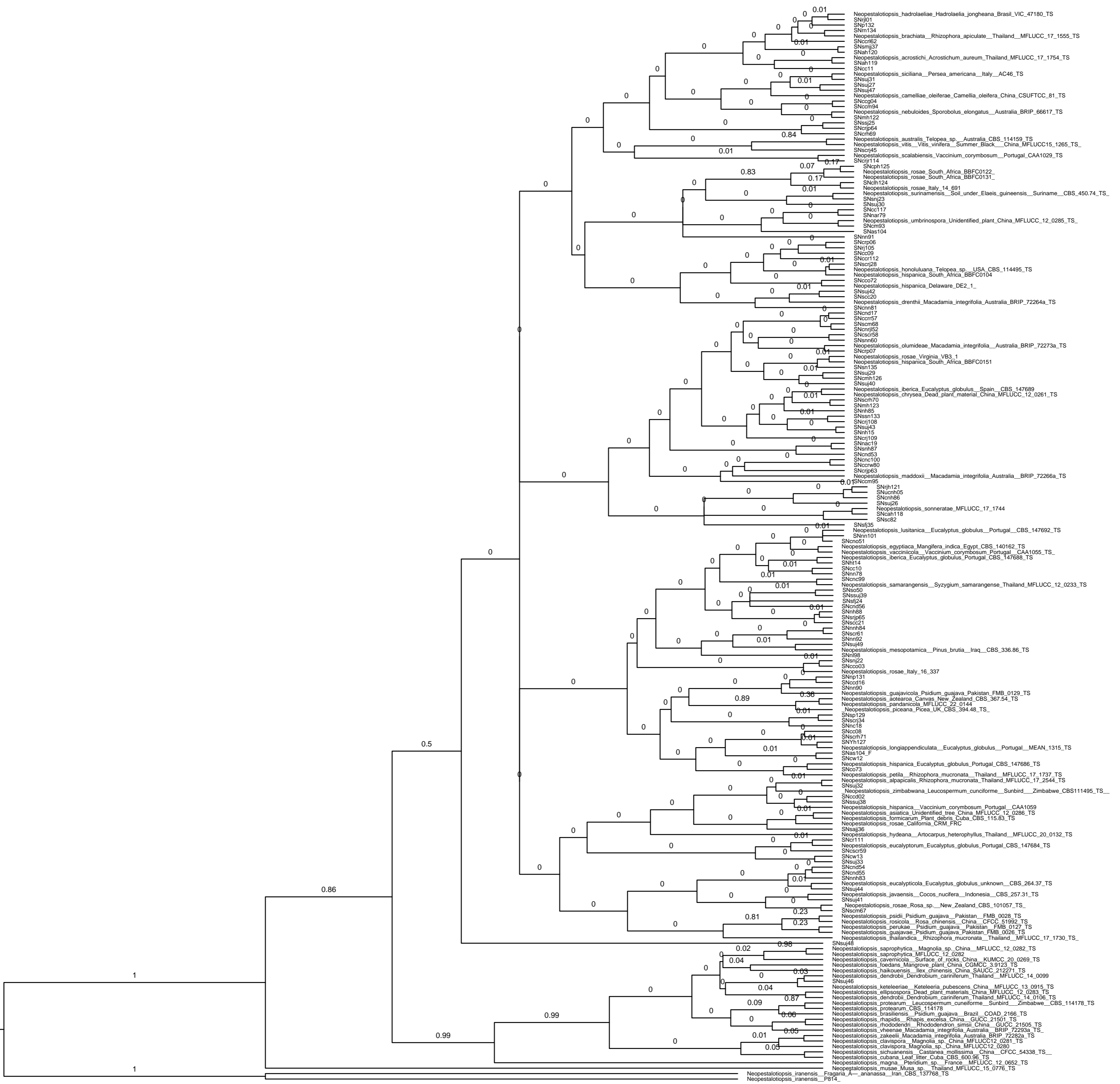

8.0E-4

Supplement: Supplementary file 1 [file pathogens-15-00010-s001.zip › Supplementary Figure S1_ITS.pdf]

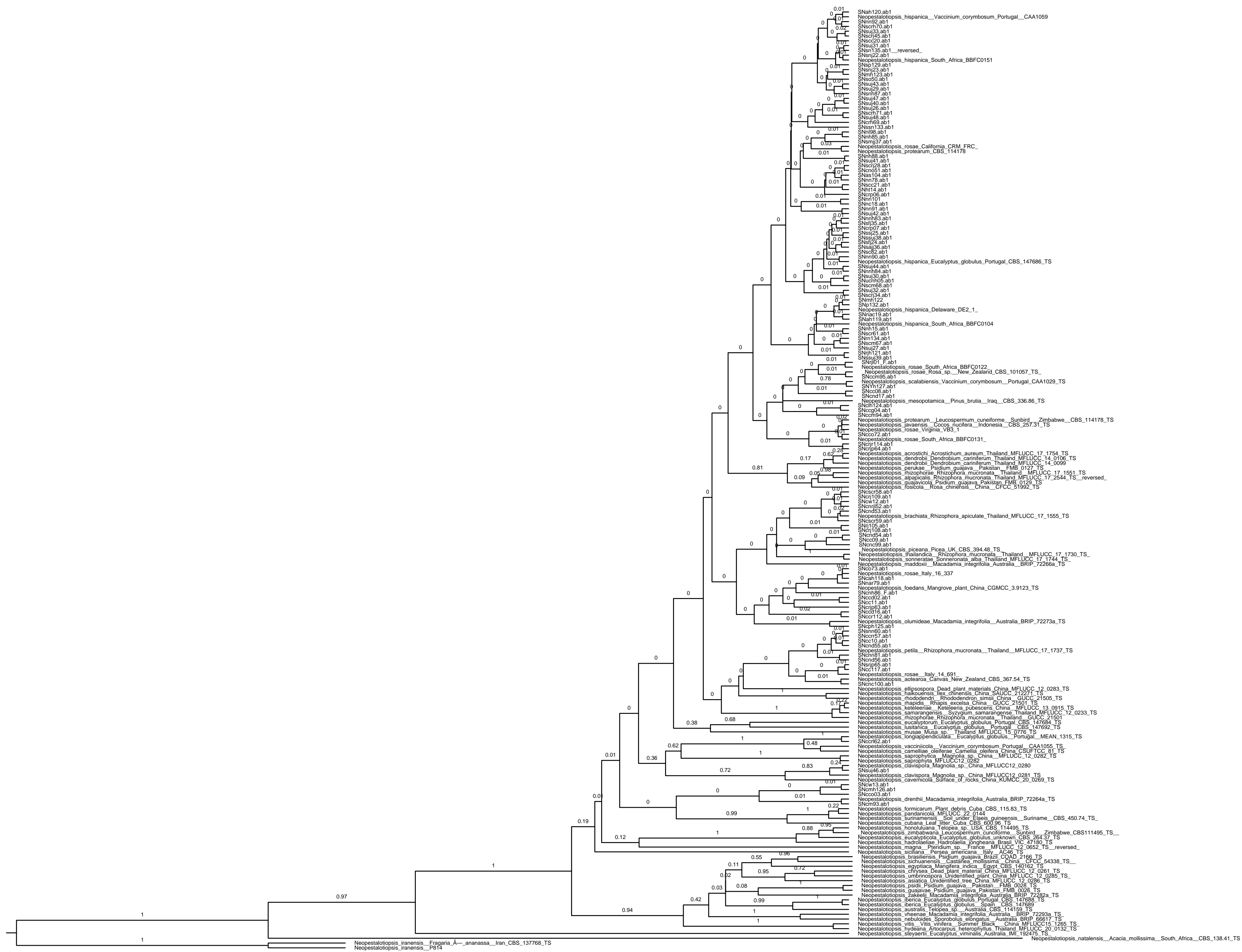

Supplement: Supplementary file 1 [file pathogens-15-00010-s001.zip › Supplementary Figure S2_TEF.pdf]

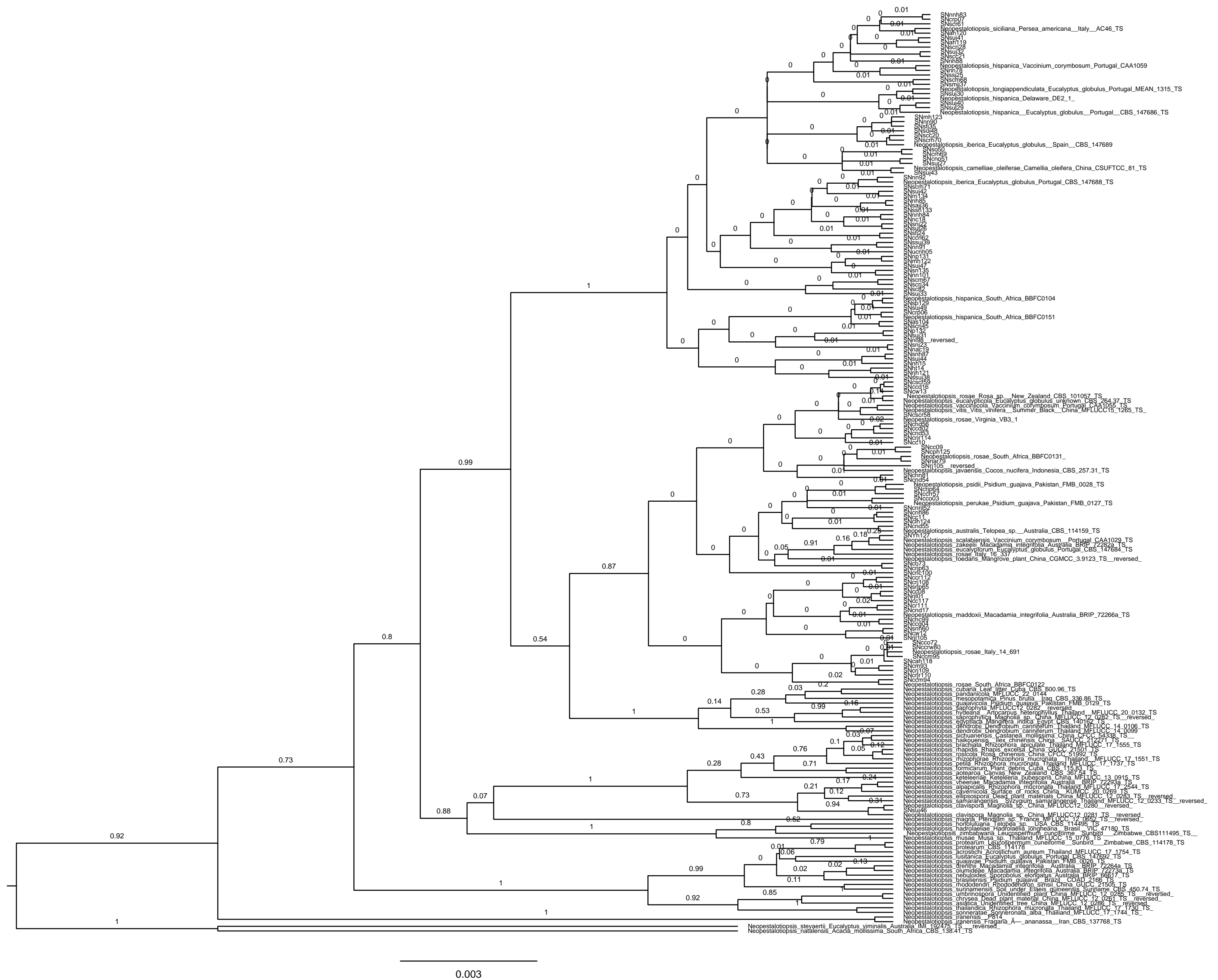

Supplement: Supplementary file 1 [file pathogens-15-00010-s001.zip › Supplementary Figure S3_TUB.pdf]
