# Supplementary material for: Identification of Neopestalotiopsis spp. from Strawberry Leaf, Fruit, and Crown Tissues in North Carolina
Source: Pathogens. 2025 Dec 21;15(1):10. doi: 10.3390/pathogens15010010 (PMC12844628; doi:10.3390/pathogens15010010)
Supplement: Supplementary file 1 [file Supplementary Figure S4_All Data.pdf › Supplementary Figure S4_All Data.pdf]

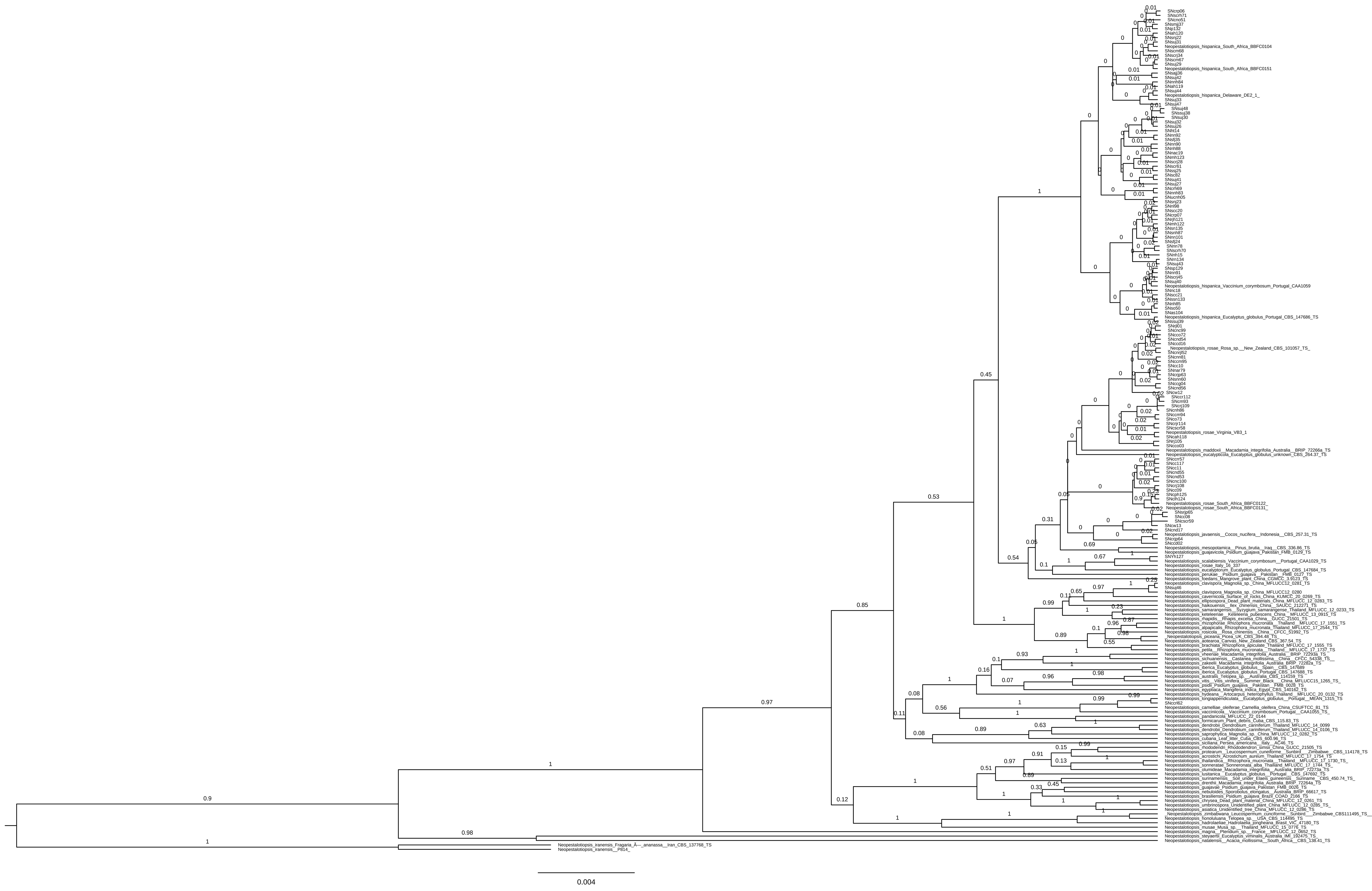
0.004
